# A General and Particular History of Anomalies of Organization in Man and Animals, Comprising Researches into the Characters, Classification, &c. of Monstrosities

**Published:** 1839-07

**Authors:** 


### BRITISH AND FOREIGN MEDICAL REVIEW,

FOR JULY, 1839.

### PART FIRST.

### Analytical and Critical Reviews.

### Art. I.

*Histoire générale et particulière des Anomalies de l'Organisation chez l'Homme et les Animaux, ouvrage comprenant des Recherches sur les Caractères, la Classification, etc. des Monstruosités.* Par M. Isidore Geoffroy Saint Hilaire, m.d., &c. &c.—*Paris,* 1832-36. Trois Tomes, avec Atlas. 8vo, pp. 746, 571, 618.

*A general and particular History of Anomalies of Organization in Man and Animals, comprising Researches into the Characters, Classification, &c. of Monstrosities.* By M. Isidore Geoffroy St. Hilaire, m.d., &c. &c.—*Paris,* 1832-36. 3 Vols. 8vo, with an Atlas.

Although some years have elapsed since the greater portion of the work before us was published, yet, as no sufficient account of it has hitherto appeared in our language, we think it important that our readers should no longer be deprived of the highly interesting and valuable information it contains, and shall therefore now present them with an analysis of it. In executing our task we shall confine ourselves almost exclusively to the exposition of the author's views; introducing our own opinions but sparingly, even when they are at variance with those maintained in the original treatise. On some future occasion we may take up the whole subject fundamentally; our present object being rather to supply facts than to criticise doctrines.

The object of M. St. Hilaire's work is to give a complete history of the subject of monstrosities: under which name all the various congenital irregularities of form and structure, occasionally met with in man and animals, are generally included. The author has collected together a great number of facts, relating to the different forms and degrees of anomaly, which he has systematically arranged in classes, orders, &c.; and he has afterwards endeavoured to establish the laws and general relations to which all the individual facts may be referred. He has shown how these laws and these relations are themselves only derived from the common laws of organization, and how, among the numerous theories of the formation and growth of animals which have been proposed in modern times, those which are not applicable to anomalous cases are also inapplicable to normal facts in general, and ought to be rejected; and many principles, on the contrary, but slightly established at present by the study of natural facts, find in the phenomena of monstrosities complete elucidation. M. Isidore St. Hilaire has also pointed out that this subject embraces all the conditions of organization in the various classes of organized beings, and that there is scarcely any general fact, any anatomical or physiological law, on which it does not throw light and either confirm or disprove. Thus the necessary consequence of an exact and profound knowledge of anomalies will be, that the study of normal and abnormal facts, intimately associated together, will lend to each other a mutual and powerful support.

A vast collection of most valuable materials on this subject may be found scattered through various publications on natural and medical science; but before the younger St. Hilaire (who has largely profited by his father's labours) undertook the task of collecting them, there existed no modern work which professed to give a complete and separate account of the various anomalies of organization, such as might serve as a text-book, and for the purposes of reference, in which all the varieties of monstrosity which have been met with should be recorded, as well as the opinions of different writers on their nature and causes.

Our author thinks that the consideration of the various kinds of monstrosity, with the laws and causes of their formation, should form a distinct branch of science, and should be treated of separately from pathological or general anatomy and physiology, embryology, or zoology; with all of which they have a very close connexion, and together with some of which they have mostly been described. To this particular subject which M. Isidore St. Hilaire has thus isolated from the sciences by which it is surrounded, he has proposed that the name of TERATOLOGY* should be given, which he considers preferable to the old denomination of *monstrosities*, the term which was previously given to all kinds of congenital malformation. Our author's views as to the separate place which Teratology should hold in science are supported by Meckel, who supposes that the various species of monstrous formation compose a series rising by regular gradations, from the natural shape to the most unnatural deformity: and that the intermediate steps are not constituted by single or individual cases, but that every variety of monstrous formation is accurately repeated in other individuals; so that, in fact, a separate and independent kingdom of monsters might be established.

Monstrosities have attracted the attention of philosophers as well as the vulgar in all ages. Among the ancients, Hippocrates, Aristotle, Pliny, Galen, and even Empedocles and Democritus noticed their occurrence and investigated their causes; and these early writers had almost as accurate notions of the nature, and gave as faithful descriptions of monstrous formations, as any of the authors on this subject before the commencement of the eighteenth century. Indeed, the history

* Derived from *τερας-ατος*, a monster, and *λογος*.

of monsters, till very lately, was composed of a collection of marvellous tales, inaccurate descriptions, and absurd and superstitious prejudices. This long period of ignorance, with respect to their true nature, may be called the fabulous period in the history of the science, and cannot be said to have terminated before the time of Ambrose Paré. A few authentic and interesting cases, it is true, had been already recorded; but these were only rare exceptions, which attracted little attention, except when some author tried to give a new and ridiculous explanation of them, derived from the fanciful ideas which were then exclusively prevalent. In fact, monsters were regarded by the writers of the seventeenth century as by those of preceding ages, as prodigies and sports of nature, arising from supernatural or unnatural causes.

After the fabulous, succeeded what St. Hilaire has called the positive period in the history of anomalies; it comprises about the first half of the eighteenth century. Evident progress now commenced, and facts were correctly observed, though still often explained on false principles. The most celebrated authors of this age on teratology were found among the members of the French Academy. Méry, Duverney, Winslow, Lémery, and Littre may be particularly mentioned. In the works of these great men, we not only find numerous facts accurately observed and described, but many judicious remarks and violent attacks against ancient prejudices. In place of those explanations of the phenomena of monstrosity, which were admitted by the superstition of the preceding period, they endeavoured to substitute scientific and reasonable theories. The causes of monstrosity particularly excited attention; and though many errors were fallen into, for want of the support of a sufficient number of facts, yet it was discovered that one of the greatest difficulties was involved in the question, whether monsters were formed so originally, or whether the monstrosity was accidentally acquired. A very long and able controversy was carried on concerning this point between Lémery and Winslow, the former of whom contended that monstrosities were formed or arose during the growth of the embryo; and modern discoveries in embryology have shown that he was correct, though his rival, who held that the germs were originally monstrous, was considered to have triumphed at the time.

The labours of these celebrated academicians conducted the science to its last epoch, which may be denominated the scientific, and which extends from the middle of the eighteenth century to the present time. It may be divided into many periods; and it will be seen that a vast difference exists between the state of teratology at its commencement and end, owing to the rapid progress which science has made. Haller may be said to have commenced this era, though Morgagni had previously corrected several erroneous opinions respecting the nature and causes of monstrous formations. Haller, in his treatise "*de monstris*," collected all the facts relating to this subject which were recorded by his contemporaries and predecessors, submitted them to a judicious analysis, and deduced from them several conclusions eminently calculated to promote the advancement of this study. Haller, however, fell into some fundamental errors, the most important of which was that respecting the mode of the development of the embryo. He supposed (and his theory prevailed till very lately, and is now partly entertained by some physiologists,) that the development of the organs of the fœtus was centrifugal, or that the heart, brain, spinal cord, &c. were formed before the vessels and nerves which were gradually developed from them. This theory, as we shall presently show, is contrary to those laws of formation by which the greater number of anomalies are explained by our author and other teratologists.

The rapid advances which have led to the present state of this department of science are owing to the indefatigable researches of modern anatomists. The study of general and comparative anatomy led the way to the true method of investigation, viz. that of comparing adult man to the embryo, and various animals to man, both in the adult and fœtal states. This comparison has given rise to two new methods of investigation, which are now almost universally recognized in the science of anatomy. One discloses the true laws of organic formations; the other embraces the general facts of the structure of animal bodies, considered in all ages and all species. Both of these methods reveal to us important knowledge concerning the composition of organs: the one plan assists us in learning the mode of their formation; and the other decomposes them by a learned analysis, and shows us the elements, everywhere identical, disposed according to invariable rules. Embryology is thus placed upon its true basis, and philosophical anatomy created. Among those naturalists to whom we are indebted for these researches, we may particularly enumerate Geoffroy St. Hilaire (the father of our author) and M. Serres in France, and Frederic Meckel and Tiedemann in Germany.

The various species of monstrous formations have been referred to three classes, viz.

1. Anomalies which arise from arrest of formation or development, in which various parts are found either imperfectly formed or altogether deficient.

2. Anomalies from excess of formation or development, in which some organs exceed their natural limits either in size or number.

3. Anomalies which result neither from arrest nor excess of formation and development, but in which the formative process seems to have been simply perverted, thus producing various modifications in the direction and situation of organs. In this class M. Isidore St. Hilaire includes the entire group of compound monstrosities, which result from the junction or fusion of two or more separate individuals. These have generally been referred to one of the previous classes.

The explanation of these different varieties of organization, or the laws of anomalies, must be derived from the general laws or principles of organization, which the study of philosophical anatomy and embryology have revealed; and, before proceeding to the consideration of the different varieties of monsters, we shall briefly mention the most interesting of these laws, and explain the manner in which they elucidate the different classes of monstrosities.

1. The first and most important is the law of *unity of organic composition.*

One great principle reigns over the whole of zoological science, that there is a unity of plan in the animal kingdom. Philosophical anatomy has shown us that the organs of animals are composed of materials which are always essentially the same, and which are combined according to definite rules; and that curious and unexpected analogies often exist between beings placed at the opposite extremities of the scale. If we admit the existence of a distinct and peculiar plan of organization for each species, or even in different families, we only obtain partial views, and the science will be reduced to the sterile observation of facts, without reciprocal connexion, rational analogies, or possible consequences. If, on the contrary, we elevate our ideas to the conception of a unity of plan pervading the whole animal kingdom, we shall only see in the multitude of beings which compose the animal series, the innumerable parts of one immense whole, the infinite varieties of one and the same type.

If we apply to the solution of the difficulties which this subject presents, the theory of inequalities of formation and development, we shall find it equally applicable to zoology as to teratology; and the fundamental truth will be apparent, that one or more metamorphoses, to a greater or less extent, sometimes consisting merely in a simple change in the mode of evolution of an organ, will explain all those varieties of form and structure which at the first aspect seem to arise from essential differences in the formative process.

The series of species in the animal kingdom seems to be parallel with the series or stages of formation or development in any individual being, or, in fact, with the series of ages in that being; and the facts of one are reciprocally connected with and explain those of the other. The connexion between teratology and zoology is now seen. The theory of inequality of formation and development relates both to the series of ages in the embryo and the series of zoological species, as well as to the series of monstrosities: it shows the parallel relation between the first and second as well as between the first and third; and by the same laws the series of zoological species and monstrous cases are necessarily analogous and parallel to each other. Thus, in an abstract point of view, all the differences between beings either normal or abnormal may be embraced in the same considerations and referred to the same formulæ: as, for instance,—the inferior beings are, as it were, the permanent embryos of animals higher in the scale; and, reciprocally, the superior beings, before they arrived at the definite forms which characterize them, have transitorily offered those of the lower animals. This must not be taken, however, quite literally; for the resemblance or analogy is only seen between individual organs, not entire beings.

By this law of unity of type in the formation of animals (which has been so fully exposed in the works of the elder St. Hilaire, Meckel, and Serres,) may be explained the resemblances which have so often been observed between the anomalous states of one species and the natural form of another. Every animal in whom there has been arrest of development should realize in some of its organs the conditions met with among the inferior classes. Excess of development, on the contrary, should cause a resemblance between the animal which is the subject of it and some of the beings higher in the scale. Many examples of monstrosity have been brought forward in support of this theory, and we may briefly state a few of them. The most numerous cases are those in which the higher animals by arrest of development present the characters which are natural to some inferior species. Thus man, when affected with monstrosity, often has a marked resemblance in some characters with different mammalia, as by the persistence of the tail,* and by many anomalies in form either of the limbs, body, or head. Thus, by the existence of a cloaca, labial fissure, duplicity of the uterus, smallness of the brain, and absence or imperfect state of the convolutions, the malformed human fœtus presents characters which are all found existing naturally in various species of *rodentia*, as the beaver, &c.

In some monsters there has been found bifurcation of the glans penis or clitoris, and two vaginæ, a disposition of parts existing normally among marsupial animals. By imperforation of the vulva, and a separate termination by distinct orifices, of the sexual and urinary organs, with imperfect development of the eyes, the genus called *aspalasomus* and other monsters realize in man those organic conditions, which in the normal state distinguish the mole and some other *insectivora* from all other mammalia. In the genus of monsters, *phocomeles*, the limbs are shortened, the hands and feet appearing to exist alone, and to be inserted immediately on the trunk, as in the seals and the herbivorous cetacea. In the rare monster, *ectromeles*, the limbs are nearly or altogether deficient, as in the ordinary cetacea.†

We may also often observe some of the conditions of animals still lower in the scale, realized in human monsters; thus, there may be a rudimentary state of the palatine arch, as in fishes; imperfect development of the diaphragm, as in all oviparous animals; a communication between the different cavities of the heart, as in reptiles; an absence of the brain and spinal marrow; and a nervous system composed only of ganglions and nervous filaments, as in the articulated animals.‡

Although the cases are much more rare in which the inferior animals resemble the higher, from excess of development,§ yet many instances of this kind have been met with. St. Hilaire has seen several individuals among the carnivora in which the tail has disappeared, and the spinal marrow has ascended in the vertebral canal, as it does in man and the most highly-organized quadrumana. This anomaly also realizes the conditions met with in some animals much lower in the series, as the anourous batrachians or frogs, where there is a continuance or excess of the process of development in the change from the tadpole to the perfect animal.

The possibility of referring the various species of monsters to a common type is a necessary and easy deduction,—in fact, an indispensable conclusion to be drawn from the theory of the unity of organic composition. When we admit that the entire classes of the animal kingdom are established upon one and the same plan, it becomes absurd to allow

* In the early stages of formation of the human fœtus, there naturally exists a prolongation of the coccyx, which is removed by the progress of development.

† It is proper to observe, however, that some of these cases were probably instances of "spontaneous amputation," and cannot, therefore, be properly called monsters at all. REV.

‡ In the last of these cases, no real analogy can be said to exist.—REV.

§ These anomalies appear to be more rare, perhaps, than they really are, on account of the much greater number of monstrosities that are observed and examined in man than among animals.

the existence of many types in one family. From the natural relation which exists also between the different degrees of monstrosity and the links in the animal chain results a complete demonstration that monstrosity is not a blind disorder springing from freaks of nature, but a particular class governed by constant and precise rules, and capable of being systematically divided into definite tribes and genera. The elder St. Hilaire, however, is disposed to consider each individual monster as constituting in itself a distinct species; and he does not agree with Meckel, that every variety of monstrous formation is accurately repeated in other individuals.

2. The second law which we shall mention as being closely connected with teratology is one of the fundamental principles of embryology: the basis, in fact, upon which that science rests, viz., that no organs originally preexist in the ovum, but are all formed at various periods of its growth. Necessarily very minute and simple at the time of their early origin, the different organs afterwards pass through a series of changes in the process of development. These changes are far from being equal either in number or importance, whether we compare together the same organ in different beings, or different organs in the same being; so that, when arrived at their definitive or permanent state, some have passed through a greater number of phases, and have departed much more from their primitive conditions than others. Such is the normal but not the invariable mode of development: an organ may stop beneath its ordinary degree of perfection, or even be entirely abortive; it may, on the contrary, exceed the natural term of its evolution, and thus will arise the two groups of anomalies, opposite in their conditions of existence, and also in their causes, to which so many of the species of monsters have been referred, viz. arrest and excess of development.

The admission of the law of non-preexistence of organs in the germ is fatal to the doctrine of original monstrosity existing before fecundation: a doctrine conceived by Licetus and the older writers on this subject, but which owed its celebrity to its adoption by Winslow and Haller. It would now have been almost forgotten had not Meckel lately attempted to revive it for the purpose of explaining the occurrence of certain monstrosities, the origin of which cannot be understood in the present state of teratology, such as the retroversion of the abdominal limbs and some other peculiarities of organization, which are constantly associated with the junction of the legs in the monsters named *symeles*. M. St. Hilaire says that the only argument brought forward by Meckel in support of his hypothesis is the impossibility of finding a satisfactory explanation of these anomalies by the theory of accidental production of monstrosities: this is true in the present state of science; but there is no reason why the obscurity of this case should not one day be cleared up, like many other facts in teratology, which were formerly thought inexplicable, and cited as certain proofs of the original production of monstrosities, but which the ulterior progress of science has discovered to be in support of the inverse theory.

According to the law which admits the formation and not the evolution of organs, monsters from arrest of development may be considered in some respects as permanent embryos: they show us at the termination of intra-uterine life some of their organs in the simple state in which they were first formed; as if nature had stopped in her course for the purpose of allowing us the opportunity of observing her processes.

3. A third law is that of eccentric development. We have already remarked that Haller (and he was followed by all the anatomists of the eighteenth century) considered that the heart was formed before any other organ, and was itself the origin of all the others; that it furnished the principal vascular trunks, which afterwards subdivided into branches more and more minute. In the same manner the nervous trunks were considered to derive their origin from the cerebro-spinal axis, which was said to be first developed, and the larger nerves were afterwards thought to ramify into the minute branches; in other words, all the vessels and nerves, subdividing more and more, proceeded from the central parts of the nervous and vascular systems towards the organs placed on the surface of the body, to which they gave nourishment and life. This theory is denominated that of *centrifugal development*, and has still many supporters.

The inverse doctrine, that of *eccentric* or *centripetal development*, was proposed by M. Serres, and is warmly supported by Geoffroy St. Hilaire and his son: all the laws of teratology proposed by the father and followed by his son in the present work are founded upon it, and by this theory a great number of anomalies are explained. These anatomists say that the vessels and nerves are formed before the heart and nervous centres; they first originate in the superficial organs on the surface of the body, and are gradually developed towards the centre; in support of this opinion it is said that the heart, brain, and spinal cord have all of them been found wanting in different monsters, while the vessels and nerves have never been seen wholly deficient. The large trunks are also found more frequently irregular in their course and distribution than the superficial branches of an artery or nerve, and the contrary should be the case if the development was centrifugal, as it has been observed that those organs which are latest formed are the least constant.

According to the observations of M. Serres, the development of the body commences on the surface of the two lateral halves, each central and single organ being originally double, its right and left portions are at first distinct and separate, and become afterwards united. If by any causes, as arrest of development, the union of these two half-organs is prevented from taking place, if this primitive state of formation becomes permanent, two lateral organs are formed, which may be either entirely distinct or only partially separated, according to the period of formation at which the arrest of development took place. The median labial fissure (often confounded with the lateral fissure or true hare-lip) has been thus explained, as well as fissure of the palate, scrotum, urethra, and spinal fissure or spina bifida, &c. M. Serres also states that the hollow organs situated in the median line are composed originally of two halves, as well as the solid organs; and his observations have been, to a certain extent, confirmed by Dr. Allen Thomson, and others. Thus, according to our author, there are at one period two hearts (this organ is placed in the first instance in the median line), two aortæ, two vaginæ, uteri, bladders, &c. These organs are considered to pass through three successive stages in the process of development: in the first they are completely double, and the two portions quite separate; in the next stage they approach and unite in the median line, the two inner walls being applied against each other, and at the third period they become definitely fused, the inner walls being removed, and all traces of separation lost. If by arrest of development the second stage of formation becomes permanent, the inner walls of the primitive organs which unite together and form naturally a temporary septum are not removed, and the organ is intersected by a longitudinal partition. Such an anomaly is sometimes met with in the human subject, affecting the vagina and uterus, and realizing the natural conditions of the sexual organs in some marsupial animals.

Another fact which is dependent upon the law of centripetal development is the greater constancy of form in those organs which are of early formation than in those later developed. When any cause comes into action at any period of uterine life, by which the process of growth may be disturbed, those organs which are already nearly or fully evolved will necessarily be little or not at all altered; but a very marked change, on the contrary, may be effected in those parts which are very imperfectly developed, or whose formation has not even commenced. In the latter case complete atrophy may be effected.

If we add, that in most of the systems of organs the different parts are subordinate in their formation one to another, the second being produced by the first, the third by the second, and so on, we shall see that the suppression of any one of them, without having any influence on those which preceded it, will necessarily cause the complete absence of all those which ought to have followed it in the order of development. The results of observation perfectly confirm these remarks; it has been found that the umbilicus and small intestines are the parts most constant in monsters, and also the organs first formed in the embryo; the spinal cord also is less often wanting than the brain which it precedes, the aorta than the heart, &c. The superficial and lateral parts of the body are also much more constant than the central or medial organs, they often exist when the latter are wanting, and they frequently present a regular conformation when the latter are seriously modified or very incomplete. Many cases may be met with where the different parts or organs have been reduced to their external covering or integument; thus in the monsters named *cyclocephali* and *otocephali*, in which the two eyes or ears are in contact, or united in one, the nose is entirely rudimentary, the bones, &c. being deficient, and only the skin remaining, which is sometimes prolonged in the form of a snout or trunk: sometimes one of the abdominal limbs has been found in this rudimentary state, and in some very imperfect monsters the whole being seems to be reduced to the tegumentary covering, inclosing a few unconnected parts, as bones, vessels, &c.

We are far from agreeing with M. Serres and our author in all the points to which this law has been applied. The centripetal mode of development no doubt obtains to a great extent; for instance, it is now generally considered that the formation of the body goes on from the lateral halves towards the centre, and that the median single organs are originally double;* but how can the mode of growth of the limbs be

* Velpeau and some others still affirm, however, that the median line is first formed.

explained on this principle? They bud out from the trunk, and are first formed beneath the skin which they reach, pushing out like little globular shoots. Again, it is far from proved that the nerves and vessels are formed before the heart and brain;* it is, perhaps, more probable that they are naturally evolved together; the existence of arteries and nerves in a monster who is deprived of heart, brain, and spinal cord, only shows that these parts are independent of each other, and that the formation of one has been arrested without affecting the development of the others.

Burdach says that the centripetal and centrifugal modes of development both obtain in distinct parts; thus, in the bones of the head and trunk, ossification proceeds from the circumference towards the median line; but in most of the bones of the extremities this process extends from the centre to the surface. The vesicula umbilicalis again enters from the surface, and forms the digestive tube, the development of which is therefore eccentric, but afterwards the salivary glands, the liver, lungs, &c. proceed from the digestive canal which they surround.†

The theory of arrest of development does not include all the phenomena of monstrosity; it throws much light upon the origin of monsters by default, but hardly any upon those by excess. Further researches upon embryology, and especially the study of the mode of formation of the vascular system, seemed to have revealed an important law by means of which many of the monstrosities by excess might be explained. It has been found that when an organ is double, the vascular trunk which nourishes it is so also; likewise the absence of a part is necessarily connected with that of its artery. This was explained by the theory that the vascular system presided over the formation and evolution of all the other organs, so that deficiency or atrophy, duplication or hypertrophy, of any organ or region was owing to a similar state of the blood-vessels, and especially the arteries.

This ingenious theory was brought forward by M. Serres, and was supported by the elder St. Hilaire, but has never been generally adopted by anatomists, for, as it has been remarked by Béclard (*Lectures on Monsters*), "it is extremely difficult to decide, in this connexion of phenomena, which is the cause and which the effect; for there is no proof whatever in support of the opinion that the development of the organs depends on that of the arteries, which is not equally applicable to the supposition that the size of the arteries depends on the volume of the organs; and that when the arteries are wanting altogether, it is because the organs which they supply are not evolved." These objections are now admitted by M. Serres himself, who has been convinced by further researches in embryology, that he must considerably restrict his ideas on this subject; and the relation, which he now admits, between the anomalies of organs and the formation of their vessels is rather that of simple coexistence than of cause and effect. In fact, according to the law of centripetal development, the organs are formed before the vessels which are distributed to them, which cannot therefore be the agents of

* The first rudiments of the sanguiferous system certainly consists in the vascular area, but the simple tubular heart is the next part which appears. The spinal cord also is the first portion of the nervous system that can clearly be discerned.

† This is not correctly expressed. The vesicula umbilicalis does not enter the embryo. The yolk-bag, its analogue in birds, does enter the alimentary canal.—REV.

their formation, though they react upon them afterwards, and perform an important part in their ulterior progress.

4. Another law by which the production of some anomalies has been explained is that of compensation or the *balancement* of organs. By this principle, as maintained by Geoffroy St. Hilaire, exuberance of nutrition in one organ is supposed to involve, to a greater or less extent, the total or partial atrophy of some other organ, and vice versâ.* It is said to be a consequence of this law that, while in the higher orders of animals, and particularly in man, some organs reach the *maximum* of development, others remain in a *medium* or *minimum* state. Many examples of this might be brought forward; thus, while man is remarkable for the size of his brain, he is equally so by the smallness of his face. Many animals occupying a low situation in the general scale of organization are furnished with some organs in a higher state of development than is met with among the superior beings: thus some of the double and lateral parts which always remain separate in the human subject, as the kidneys, approach and unite by a continuance of development in fishes and many aquatic birds. The two eyes naturally coalesce into one in some of the lower animals, as the *monoculi* and other entomostraca; and these states of organization, which are normal in these creatures, are sometimes imitated in human monsters, and must then be considered to arise from a true excess of development; though this excess, as in the case of junction of the two eyes, cannot take place without a corresponding atrophy or suppression of some parts, and a marked arrest in the development of many others. This compensation or balancing between the different organs in the monster only renders the analogy between the malformed human fœtus and some of the inferior animals more complete, for the defects and excess of formation coincide in the normal cases as well as in the abnormal.

Innumerable applications of the law of compensation may be made to the study of monstrosities; thus, in an individual who has several supernumerary fingers or toes on one hand or foot, we often find the opposite limb with less than their natural number. Andral says that supernumerary fingers are often found in those monsters in which other parts of greater or less importance are incompletely formed or altogether deficient, as in cases of *cyclocephalus.* Two distinct anomalies of size, the one by diminution and the other by increase, may occur together in the same subject, in consequence of this antagonism of development; thus, one kidney has been found extremely small or almost atrophied, and the other in a state of hypertrophy, or the kidney has been seen very diminutive, while its capsule was very voluminous. Cases which might be cited in support of this law are met with every day.

5. The next law of organization which we shall mention, is that of similar position or affinity of similar parts for each other ("de soi pour soi.")

When two or more organs perfectly resemble each other, they seem to have a tendency to approach and unite. This has been observed be-

* The *fact* of the compensation of organs was long ago recognized by Paley, who pointed out that exuberant nutrition of one organ is generally accompanied by diminished size of another. We do not feel inclined to accord with M. St. H. in regarding the former condition as the *cause* of the latter.—Rev.

tween similar organs and similar portions of organs in one individual, as well as between two distinct individuals. Many anatomists at different times, when examining cases of double monstrosity, have been struck with the remarkable relation of situation and connexion which the two subjects offered towards each other; but it is only within the last few years that this circumstance has received much attention, and been studied in a philosophical point of view. The regularity of disposition which two beings present when united together is not a rare circumstance, a peculiar characteristic of certain monsters, but is constant and common to all, and must be considered as a fact of high importance, influencing all the other facts of double monstrosity. The two subjects which compose a monster completely or partially double, are always united by corresponding aspects of their body; that is to say, side to side, face to face, or back to back. Each part and each organ in the one corresponds to the same part or organ in the other; every vessel, nerve, or muscle, situated in the line of union, joins itself to the same vessel, nerve, or muscle, in the other subject, in the same manner as the two primitive halves of any single organ, which are originally separate, unite by the progress of development.

These general facts, very important in themselves, are equally so from the numerous consequences which may be deduced from them; thus they serve to confirm the proposition, that the organization of monsters is governed by very constant and precise laws, and that all the irregularities of monstrosity never break through certain limits. By a knowledge of this law it becomes possible, when reading the descriptions and looking at the figures of monsters in old works, to distinguish those monstrous combinations which might really have existed, from those which are only the fanciful and absurd productions of imposture or of a fertile imagination.

The important law of affinity of similar parts for each other—established by the elder St. Hilaire, from researches made on anomalies of the most complex description, and verified, as we have shown, in all cases of double monstrosity—is also fully confirmed by the study of the simplest deviations of form and structure. We shall see this great principle account with equal facility for the union of two organs, two systems of organs, or two entire individuals. It is by this principle that we explain the different anomalies by junction or fusion; or, as they are denominated by M. Breschet, *symphyses*. Thus the two kidneys, the two eyes, the two ears, may join, and form a double kidney, eye, or ear, or the two organs may unite and form a simple or single organ, in which no traces of duplicity remain; in the same manner as in the embryo the two primitive uteri join to form a single symmetrical uterus, which union was observed to take place by M. Serres, when making those researches upon which he founded the theory of eccentric development. These anomalies, by the junction of two lateral organs in the median line, are the consequence, as we have previously remarked, of excess of development. The theory of eccentric growth shows us that single symmetrical organs, when divided, are in their primary state of development, and, when united, in their secondary degree. The eyes, ears, kidneys, &c. naturally stop at the first stage, and only accidentally arrive at the second, when they present anomalies by fusion.

If we consider that the anomalous states, by junction of double organs, arise from the same laws which regulate the natural union of parts, and are only particular effects of one universal cause, we can easily conceive that similar parts ought to be exceedingly subject to be joined and confounded together; and deviations of this kind are, in fact, very common, for there are scarcely any double organs which do not furnish us with examples. Many of these instances, it is true, are met with in cases of great monstrosity, because most of the lateral organs are separated from each other by many important parts, as in the eyes and ears, and cannot unite in the median line without the suppression of these parts, but union may take place between the kidneys without any serious alteration even of connexion. Junction of the testicles, ovaries, ribs, teeth, fingers, &c. must all be explained by this law.

We must here mention another fact, which, on account of its general application, and the light which it throws on the study of anomalies, might even be elevated to the rank of a separate law. Those parts which are several times repeated or multiplied in the body, which, in other words, are homologous, or have many congeners, as the fingers, toes, teeth, &c. taken together, form organs very important, and very constant in their development, but each part individually is of little relative consequence, and subject to vary, the frequency of which variation is proportionate to the number of analogous parts. Almost all the incontestible examples of the addition of really supernumerary parts have been met with among those organs which have the greatest number of similar parts placed in series, as teeth, &c.; and that this multiplication of organs sometimes really takes place must be admitted, though this species of excess of formation is much less common than has generally been believed. In fact, the greater number of anomalies which have been referred to this head, arise from causes altogether opposite, as in the entire group of compound monsters, which are caused by the junction or fusion of two distinct beings, and not the addition of supernumerary parts to one individual. Many simple anomalies also, in which the number of organs is apparently increased, only arise from the division or separation of one part into two, the primary state of formation having remained persistent, and thus these malformations must be referred to arrest instead of excess of development. We may here remark that the organs which are most subject to numerical variation, are precisely those which are found to be least constant in number in the different genera of animals, such as the teeth, vertebræ (except cervical), ribs, fingers, &c.

In the foregoing view of the different laws of organization which have been created, we have simply stated the opinions of the writers by whom they have been formed, and we are far from admitting all the conclusions which have been drawn by them, (in many instances too hastily, and from too small a number of facts.) In the first place, it has been lately said,* that one of the fundamental principles by which the formation of a great number of anomalies has been explained is in many cases quite unestablished. We allude to the theory of arrest of development, which, it is said, will only satisfactorily account for a very

* Cruveilhier, in the Bulletin de l'Académie Royale de Médecine. Tom. iii. Nos. 4 and 5, Dec. 1838.

limited number of malformations; for before a congenital lesion can be stated to result from a stoppage in the process of growth, it is necessary to ascertain whether any of the transitory states of fœtal life correspond with this lesion.

The researches of embryology have shown that the product of conception, between the first period of its formation and the time of its complete development, passes through a series of changes as wonderful as the metamorphosis of insects; and the comparative study of these temporary states of the human fœtus with the permanent forms of the inferior animals teaches us that the former often represent the latter; and since in a few cases certain vices of conformation have been found to represent, both some of the temporary fœtal stages and some of the permanent states of the lower animals, generalizations have been hastily formed, and conclusions frequently drawn, which are supposed to apply to all cases, without any further examination being made. The great error has thus been fallen into, of not requiring direct proof, but of allowing *a priori* conclusions to take the place of reasonings *a posteriori*, upon which alone theories should be founded in science. We should never be contented with any presumptive evidence derived from induction or analogy, however ingenious, but we must seek for direct proof; and before we explain the origin of any malformation by arrest of development, it is necessary to show, by anatomical investigation, that. the embryo presents a similar disposition at some epoch of its life.

Many anomalies included in the great class of congenital adhesions (*symphyses* of M. Breschet), as the occlusion of some of the natural orifices of the body, the anus or vagina, for instance, have been referred to arrest; but, except in the case of the pupillary opening, Cruveilhier says that it has never been shown that any of the natural apertures are closed by a membrane at any period of uterine life. We will take another class of malformations, viz., congenital solutions of continuity, which have been considered to arise from the persistence of certain conditions peculiar to an early state of fœtal existence, and not as any new or accidental state. M. Cruveilhier says,* that if this is the case, this stage of formation must exist previously to the fifth or sixth week of gestation; after which period, he adds, "I can certify that it will not be met with." He also says, "I avow my incompetence to detect the structure of the human fœtus before the fifth week, and I have a great distrust of any observations made on an embryo, the formation of which has scarcely commenced; for since it is even difficult to see parts which are considerably developed, it must be far less easy to study the rudimentary states of microscopic organs, so delicate, that not merely the least touch, but even the movements of the liquid in which they are placed for the sake of examination, is capable of tearing and destroying them."

As in the case of obliteration of the pupil, there are some instances of separation of parts naturally united, which must be referred to true arrest of development, as persistence of the arterial and venous ducts and foramen ovale, permeability of the umbilical artery or vein, or of the urachus; also, persistence of the communication between the peritoneum

* Ibid.

and tunica vaginalis; but, with the exception of a few cases of this kind, it has been said* that none of the other anomalies belonging to this class can be referred to corresponding states in the life of the fœtus; thus can hypospadias, hare-lip, spina bifida, &c. ever be observed as natural conditions of the embryo, and have they not been considered as effects of arrest of development upon insufficient evidence? The remarks which we have made concerning this theory also apply in many respects to some of the laws of development; and particularly, as we have before stated, to that of centripetal formation, which cannot be verified in all parts by an examination of the mode of growth of the organs of the embryo.

Before we proceed to consider the different groups into which anomalies have been divided by our author, we shall say a few words on the very obscure subject of the causes of monstrosity, the original agent which produces a modification in the formative process. The difficulties which here surround us, are common to this, with all the other departments of natural science. Nature only presents effects to our observation, or at most their proximate causes, and the primary or directing influences can only be deduced from these effects. Teratology is so closely connected with embryology, the laws of one being derived from those of the other, that, while the causes influencing the original phenomena of normal development are unknown, those presiding over irregular formation must necessarily be involved in darkness; and direct observation is far from having furnished us with the results which might have been expected concerning the early stages of gestation, the conditions of the very young embryo, and the first steps of its development. The ancient writers took much interest in this mysterious subject, and mixed up many ingenious but fanciful ideas with the most unnatural absurdities. They almost all agreed in one point, that monstrosities arose from some disturbance or imperfection in the act of fecundation. In the opinion of some, however, malformation or disease (as tumours) of the uterus of the mother was capable of producing deformity of the child; and, lastly, unnatural connexions were admitted by all the older physiologists to be incontestible causes of monstrosity.

Most of these notions were founded on the hypothesis of original malformation of the germ; but now that this theory is discarded, we must look to influences operating after fecundation, during the progress of development. In the first place, the birth of a monster has sometimes undoubtedly followed various accidents received by the mother during the early months of gestation, as a fall, violent blow, &c. A vivid mental emotion will occasionally have the same effect, or long-continued anxiety of mind; and in proof of the action of the last cause, it has been observed that unnatural productions are more common among unmarried than married women.

Direct evidence that the development of the embryo may be affected by external causes has been afforded by experiments performed on the eggs of birds. Geoffroy St. Hilaire allowed the process of incubation to

* Ibid. Cruveilhier has been here a little too sceptical; it is, certainly, well established, that at one period of fœtal life all the natural apertures of the mucous canals are closed.

commence in some eggs under ordinary circumstances; and after it had proceeded naturally for a short period, about three days, he performed various experiments on them, as shaking them violently, perforating them at different points, keeping them in a vertical position, upon either the large or small end, covering a part of the shell with wax, or some varnish impervious to the air, &c. The effect of these perturbations was the constant production of a large number of monstrosities. These experiments were repeated by the younger St. Hilaire, in a different manner. He altered the structure of the eggs before the process of incubation had commenced, and not as in the previous instance during its course. Entirely different results followed; for in the latter case no anomalies were ever produced, vitality was mostly destroyed, the embryo in some cases seemed retarded in its development, but was never monstrous. The opposite effects of these experiments show the truth of the proposition, that the origin of anomalies is accidental, and not primitive.

Another of the most generally admitted causes of malformation is disease of the embryo itself. Haller and Morgagni among the older writers, and Meckel, Béclard, M. Dugès, Cruveilhier, &c. in recent times, have especially adopted the theory that a great number of the anomalies of the superior regions of the body may be explained by the occurrence of hydrocephalic disease. It has been said, especially by Béclard, that dropsy is an affection to which the fœtus is more liable than any other, and that the brain and parts connected with it, as the spinal marrow, are especially prone to be attacked. If this disease comes on at an advanced period of utero-gestation, it may occasion separation of the bones of the cranium, if earlier, it may produce hydrocephalic hernia and spina bifida. The protrusion of the brain may remain till birth, or it may have disappeared, causing destruction of the cerebral masses and vertebral cord; in which case, an *anencephalous* monster will be produced, in which the cranium and spinal cord are widely open and their contents deficient. By the action of this and other diseases many malformations have been explained, and this theory is doubtless partly true, though its advocates have carried it to far too great an extent.

Geoffroy St. Hilaire has proposed another hypothesis, by which he accounts for the production of many monstrosities. His idea is that unnatural adhesions sometimes take place between the embryo and its membranes, occasioned, perhaps, by some accident which tears the membranes, and probably allows of the escape of part of the contained fluid; when the edges of the aperture coming in contact with some of the organs of the fœtus, union takes place, and bridles and false membranes form, which may mechanically obstruct the development of different organs, causing arrest of formation or distortion, as in the case of club-foot. St. Hilaire artificially produced this connexion between the embryo and the shell in birds, by unnaturally raising the heat during incubation. This morbid change may account for some forms of malformation, as unnatural connexion between the placenta and different parts of the fœtus, which has been observed in several instances. A case of this kind was lately related by Dr. R. Lee to the Med.-Chir. Society of London, in which the placenta was attached to the integuments of the forehead by a membranous hand, three quarters of an inch in breadth and one inch and a half in length; the portion of skin where the union had taken place was preternaturally vascular, and presented the structure usually observed in nævi; the development of the skull had been arrested, and all the bones forming the vault of the cranium were deficient, but the brain itself and its membranes were healthy.

None of the explanations which we have given throw any light on the causes of compound monstrosity, which consists in the union of two or more individuals. It is an important question to decide whether the germs or ova are originally double: the most general opinion seems to be that they are not; we must therefore enquire how the junction of two separate beings can be explained. Why do not twin embryos always unite? Two perfect and distinct fœtuses have, according to our author, been occasionally met with contained in the same membranes, so that mere contact or pressure cannot account for it. The law of affinity of similar parts for each other has shown that the two individuals composing a double monster are always united by similar parts of their body; do ova then always unite when they happen to be placed next each other by corresponding aspects? This proposition requires to be verified by observation and experiment. In a remarkable memoir communicated to the French Academy of Sciences by MM. Delpech and Coste in 1832, these physiologists state that there exist two electric currents, which are directed towards the same point, in two embryos, placed opposite to each other, and opposed by similar surfaces, but which run in a contrary direction in those which are placed differently. In consequence of this, they say that the first are always united, and the latter as constantly isolated.

We have mentioned that, in some cases, monstrosity of the fœtus appears to have followed external causes which have affected the mother, as a blow or fall, and in other instances a vivid or long-continued mental impression. Some congenital malformations appear also to be hereditary; this has been particularly observed among the slighter anomalies, as albinism, irregularity in the number of fingers and toes, and hare-lip. St. Hilaire says that this last imperfection is seldom or never transmitted from parents to their offspring. The writer of this article has seen, however, several well marked instances of it; in one family with which he is acquainted, where the father has hare-lip, two of the children are affected with this anomaly, one having single and the other double lateral fissure. We have heard it stated, that in Hunter's celebrated case of hypospadias, where impregnation was effected by a syringe, the paternity was assured by the existence of the same malformation in the offspring.

In concluding this part of our subject, we may remark, that though a violent shock, or even anxiety of mind, has apparently disturbed the progress of development in some cases, yet there is no reason to suppose that any slight or momentary influence can have any such effect. It is contrary to science and reason to suppose that any object merely seen, dreaded, or wished for by the mother, perhaps during the latter months of pregnancy, can in any way affect the child in her uterus, or be depicted on its body—a prejudice which is as dangerous as it is ancient.

M. Isidore St. Hilaire divides the various kinds of malformation into four groups, which are not characterized, according to the plan of most preceding authors, by any supposed alteration in the nutritive process, as excess or default of development, but are founded on simple and obvious characters, either referrible to the degree of unnatural change or to the region or system of organs affected.

1. The first group to which he confines the term of anomalies, comprises those varieties or defects of conformation which are simple and uncomplicated, often not apparent externally, and though in most instances congenital, yet not necessarily so in all, as in the cases of giants and dwarfs, which our author has included here, and which arise from some irregularity in the growth of the body, which continues to act after birth.

2. The second group consists of those congenital changes in the situation of organs, in which the *relative* position and connexions of the parts are not altered. A great number of organs may here deviate from the specific type without the performance of their functions being in any way impeded. In man, and in all the higher orders of animals which are symmetrically formed, this anomaly is confined to transposition of the viscera; but in some of the inferior beings which are unsymmetrical, all the organs of the body are transposed: thus in those testaceous mollusca which are furnished with a spiral shell, the spire, in a case of *heterotaxy* (the name given to this group), will turn in an opposite direction to the course which it naturally follows.

3. The third group includes the various forms of *hermaphrodism*, in which both sexes are either present in one individual or some of their characters.

4. The fourth and last group comprises all those congenital anomalies which are complicated, many organs being seriously altered, both in their anatomical and physiological relations, producing great external deformity. St. Hilaire restricts the title of monstrosity to this group.

In the brief analysis which we shall now give of the details of the work, we must altogether pass over many of the classes and orders into which these groups are subdivided, and confine our attention to those which are most interesting, and which throw most light on the laws and causes of their production.

The first group, that of simple anomalies (*hémitéries*), is divided into five classes, which are based upon the kind of malformation with which the part is affected: thus, either the volume, form, structure, disposition, or even the number and existence of organs may be altered.

The first class consists of those cases in which there is general or partial increase or diminution in the size of the body. It includes all the forms of giants and dwarfs, and a great number of other anomalies which do not possess much interest, as unnatural development of muscles, extreme narrowness of the vagina, abnormal increase or diminution in the volume of the mammary gland, &c. Our author has given a long and interesting history of giants and dwarfs, to which we must refer the reader, as it would take up too much of our room to dwell upon it here, and oblige us to omit much more important matter; in fact, it is very doubtful whether the greater number of these cases can be properly included at all among true monstrosities, as they evidently arise from causes altering the process of nutrition after birth. Dwarfishness is almost always the result of disease during infancy, particularly rickets, some traces of which affection are exhibited by almost all these diminutive individuals, as unnatural size of the head, with disproportionate shortness of the legs, curvatures of the limbs, &c. In some cases, however, diminution of size seems to arise from causes producing imperfect nutrition of the fœtus in utero, as malformation of the uterus and disease of the embryo itself. Dwarfs are exceedingly rare among wild animals, a fact which may partly be referred to their immunity from rickets.

The causes which lead to unnatural increase of development and the production of giants are wholly unknown, but we may remark that individuals who exceed the usual limits of their species are more rare among animals than those of diminutive stature.

Together with the description of giants and dwarfs are included an account of many curious cases which have been met with of precocious development of the physical and moral powers, and particularly of the generative system. Many instances have occurred, in which children of three and four years old have presented all the signs of puberty. This unnatural state of the organs of generation is generally accompanied with premature increase of the general volume of the body, and in the male subject with other signs of virility, as the development of hairs, muscles, alteration of the voice, &c. When the sexual apparatus has thus early arrived at its perfect state, it frequently happens that the general growth from that time becomes retarded or stops altogether, these forward individuals not becoming giants, but even remaining below the ordinary dimensions; so that, though remarkable during infancy for their great bulk and stature, they become peculiar afterwards for the smallness of their size.

As, in those cases which prematurely arrive at manhood, growth generally stops, so it has been observed that in giants, where the process of development is carried to an unnatural extent, and continued for an unusual length of time, many of the characters of infancy are preserved to adult age, and the phenomena of puberty are imperfectly or tardily shown: so that, as St. Hilaire has remarked, an intimate relation seems to exist between the evolution of the generative organs and the general growth of the body.

The second class, which includes the various anomalies of form, is closely connected to the last, for the simultaneous existence of several partial alterations of volume, some by diminution, and others by increase of size, must naturally change the shape of organs. There is, also, a great affinity between this class and the fourth, which includes the alterations of position; for the situation of parts cannot well be altered (particularly those that are external) without changing the form of some portion of the body: thus, in club-foot there is a combination of these two classes of anomalies. Varieties in the conformation of parts are more common than any other kind of organic deviation; and there is no region or organ which may not present numerous modifications of external form. Though so frequent, these anomalies present few remarkable peculiarities, and consequently are of little interest. The alteration in the shape of the heads of idiots and cretins is an example of these malformations.

Our author passes them over very quickly, and goes to the next class, which includes the various changes of structure or intimate composition of parts. The most interesting anomalies found here are the varieties in the colour of the skin, arising from deficiency or excess of the pigment or colouring matter deposited in the rete mucosum of Malpighi. Perfect *albinism*, in which there is complete absence of the pigment, is much more frequently observed among the dark inhabitants of hot climates than the fairer races of cold countries. Albinos are most common in Africa among the negroes, and after them among the inhabitants of the isthmus of Panama. These peculiar individuals are generally of a delicate constitution and badly proportioned, their heads and hands being too large; their physiognomy is without expression and disagreeable, from the eyes being intolerant of light, half shut, and having a constant twinkling or oscillatory movement, arising from the complete want of pigment or colouring matter in them, which also causes transparency of the iris, with a red appearance of that organ and the pupil. In consequence of the iris not intercepting any of the rays of light which enter the eye, vision is very imperfect, except in the dusk. Albinos generally possess a lower degree of intelligence than the rest of their race; and among the negroes they are despised and badly treated. It has been observed among the negro race that albinism is more frequent in women than men; and females possessing this defect may produce children, by black men, which are either pied, entirely white, or wholly black. With respect to the causes of this anomaly, it must be referred to simple arrest of development; our author says,

"We know that the pigment is wanting in the fœtus up to a very advanced period of intra-uterine life, and that even in black or dark people the integument remains for some time after birth of the same colour, as in the children of fair men. We can easily conceive, therefore, how the skin can stop in the series of its stages of development, before the period when in the natural order of formation the pigment is deposited in the mucous layer, and consequently it will remain uncoloured. The colouring matter of the skin and hairs, the iris and the choroid, may thus be deficient in an individual (independently of any pathological alteration), in the same manner as any organ or part of an organ may be wanting from arrest of development." (Tom. i., p. 319.)

St. Hilaire further remarks:

"If any doubts remain regarding this explanation, I may remark that the absence of the pigment is not the only condition of fœtal life which is preserved in albinism. We know that the child, during the second half of intra-uterine existence, has the skin covered with down; this down is frequently preserved in albinos, particularly in those of the isthmus of Panama; lastly, the persistence of the membrana pupillaris, in some of these cases, beyond the ordinary term of its existence, is another equally evident proof of arrest of development." (p. 320.)

Albinism is frequently seen among animals, particularly those in domestication: white horses, pigs, rabbits, ferrets, cats, &c. are daily met with, some of which are perfect, others only partial albinos.

The presence in the rete mucosum of an unnatural quantity of dark coloured pigment constitutes the opposite anomaly to albinism, and is named *melanism.* Perfect melanism has very rarely, if ever, been observed in the human subject; no perfectly authentic instance is recorded; but among animals and birds it is commonly seen; black deer have often been found in a wild state, as well as some of the larger carnivorous animals; black sheep, rats, mice, &c. are not uncommon. Though complete melanism is unknown in the human race, partial melanism is very frequent, for to this anomaly must be referred many of the congenital spots and marks which are denominated nævi, moles, &c., and which are often confounded with the red vascular tumours or spots which arise from malformation or disease of the cutaneous vessels. These melanotic spots or patches, which are so frequently seen, particularly on the face and back, are very variable in their form, colour, and appearance; they may be elevated above the skin, or be even with its surface, smooth or covered with hairs, &c. With respect to the causes of this anomaly, it must be referred to true excess of development, arising from some unknown influences.

The fourth class of simple anomalies includes a great many interesting malformations, as changes of position, or displacement of organs, and alterations of connexion, some parts which are commonly separated adhering together, and others naturally connected remaining separate.

Organs are liable to anomalous changes of position in proportion as they are loosely connected with the surrounding parts, particularly at the early periods of their formation. Thus the walls of the splanchnic cavities are much less subject to alterations in the position of their component parts than the viscera contained within them, which float as it were loose, and some of which naturally vary slightly in situation at different periods of development. The viscera are in some cases transposed from one part of their natural cavity to another, while in other instances they may be transported into another cavity, or even become external. In the last case the anomaly is called a congenital hernia. The brain may be variously displaced in this manner, it may even pass out of the cranium through any of the sutures. M. Serres has seen the brain protrude in the median line, between the right and left halves of the ethmoid and sphenoid bones, so that some portions of it descended with its membranes, through the base of the cranium, into the nasal fossæ, and even into the pharynx. This fact is explained, according to M. Serres, by the law of eccentric development, which supposes that each organ, or part seated in the median line, is formed by the union of two lateral halves, originally distinct and separate.

The situation of the heart may be altered in various ways; one of its most interesting anomalies is where this organ is placed in front of the neck, immediately above the chest. Although rare, this malformation has been observed in man by Vaubonnais, Walter, Breschet, and others. This position of the heart realizes one of the natural characters of fishes, and is also a normal condition of the early periods of uterine life in man. It results from this displacement, that the aorta must first descend instead of ascending, and it will form no arch, which is also the case in the embryo.

With the unnatural separation of parts are included those anomalies in which various orifices and canals, usually closed after birth, remain persistent, as the urachus, ductus arteriosus, foramen ovale, &c. Cases in which the urachus has remained pervious to adult age are not very rare. When this canal only exists in part of its extent, nothing indicates the anomaly; and should it even continue to the umbilicus its presence will be unknown so long as the normal urinary passages are free; but if they become obstructed by any disease or malformation, the total or partial excretion of the urine will take place by the navel, and the defect is apparent.

The ductus arteriosus has been found in its original pervious state in many instances. This anomaly may exist independently of any other malformation of the organs of circulation, but it is generally complicated with other defects, as an aperture between the two auricles or ventricles, or even absence of the inter-auricular and ventricular septa, so that the four cavities of the heart are converted into two. The origins of the pulmonary artery or aorta may be transposed, one vessel communicating with both sides of the heart, or both vessels with one ventricle. The commencement of the pulmonary artery has even been found entirely obliterated in some cases of persistence of the ductus arteriosus.

"These different defects of conformation," says M. St. Hilaire, "have most of them been observed, independently of the presence of the arterial canal; but more than one of them usually exists in the same subject, which may be combined with any of the others in various manners, giving rise to different states of the circulating system, which resemble the various conditions of fœtal life, and, more or less exactly, some of the characters which are presented by the lower vertebrated animals, particularly reptiles." (Tom. i. p. 563.)

Any of these malformations of the heart will produce the disease called cyanosis, which results from the communication between the arterial and venous systems.

All the organs seated in the median line are liable to be separated into two lateral halves, in consequence (as it has been said) of arrest of development. The bones offer numerous examples of this anomaly. Fissure of the sternum has often been met with, giving rise, in some cases, to hernial displacement of the heart. Partial division of the vertebræ is a very common defect, and is known by the name of spina bifida. This term is generally restricted to those cases in which the fissure only extends through the posterior part of the rings of the vertebræ; the spinous processes are here mostly divided, but they may be entirely separated from the rest of the bone, and be removed to a distance, so that the posterior wall of the spinal canal is partially or entirely wanting. The fissure may extend completely through the bodies of the vertebræ, separating the right side of the spine from the left; this, however, is exceedingly rare. Spina bifida, or, as it is more correctly denominated, spinal fissure, has been almost always confounded by pathologists with hydro-rachis, or dropsy of the spinal canal; it is, however, necessary to distinguish them, for one often exists without the other. Spinal fissure varies greatly, according to the number of bones affected, and the region in which it occurs. The whole of the column, from the first cervical vertebra to the bottom of the sacrum may be open; and in this case the malformation is generally accompanied with division of the cranial bones and entire absence of the spinal cord. In the greater number of cases the fissure is only partial, the lumbar region being most frequently affected. This anomaly is often connected with some malformation of the ventral aspect of the body, as extroversion of the bladder,* eventra-

* The front wall of this organ is here deficient, together with part of the walls of the abdomen; and the mucous membrane of the posterior wall of the bladder projects above the symphysis pubis in the form of a soft red tumour.

tion, exomphalos, &c. These severe and complicated cases, however, belong to the last group, or true monsters, as many of them are incompatible with the life of the child.

Most of the simple anomalies which various organs present may be referred to changes of volume, form, structure or disposition of parts; but some remarkable cases exist in which there is not only a modification in the conditions of existence, but even an alteration in the number of parts, which may be either increased or diminished.

These numerical alterations seem, *primâ facie*, to be clearly defined, and circumscribed within very precise limits; but if we accurately examine the different cases which are comprised under this head, we shall find a great number which may be confounded with, and which even properly belong to, the preceding classes; many may be referred to alterations of volume, while others arise from unnatural connexion or division of organs.

"When an organ disappears, it sometimes happens that some obscure rudiment of it may be found, by a minute dissection, and thus it has not actually ceased to exist. On the contrary, when supernumerary parts are added to any of the natural organs, anatomical analysis is sometimes capable of showing that there is not any addition of new parts, but only an increased development of those which commonly exist in a rudimentary state." (Tom. i. p. 622.)

There are also a great many cases in which the addition or deficiency of organs may be explained by the intimate union or complete division of two or more parts; but it is very difficult, nay, almost impossible in some cases to distinguish whether there is actual absence of an organ or only the fusion of two together; and whether a part is really double or only divided.

Though many of these anomalies may be thus referred to other classes, yet some still remain which present peculiar conditions, and which seem to result either from the total suppression of some part, or, on the contrary, from the addition of others completely strange to the normal states of organization. These necessarily form a separate and remarkable order, differing essentially from all other simple anomalies, and not capable of explanation by any of the laws which are known in the present state of science: they have been considered by some as incontestible proofs in favour of the theory of original monstrosity; but, as we have shown that many of those cases which primarily seem to belong to this class must really be referred to others, the origin of which are understood, there is little doubt that by the progress of science the nature of all those which remain will be elucidated, without recurrence to the hypothesis of monstrous germs.

The bones, muscles, and other organs present many numerical anomalies. Thus the muscles of the limbs sometimes vary in number; and it is remarkable that the muscles of the arm, forearm, or hand, scarcely ever depart from their normal type by alterations in the number and disposition of their parts, without falling into the natural conditions of the thigh, leg, and foot, and *vice versâ*. An evident analogy, therefore, exists between the upper and lower extremities; and Meckel, who has made many researches on this subject, has also discovered that many relations exist between the numerical varieties of muscles in the human subject and the normal conditions of the muscular system in different animals.

In this place we may mention another curious and interesting fact, which is connected with the subject of alterations in the number of parts. It is well known that in the lower vertebrated animals, and especially the invertebrated classes, many organs will be reproduced after being removed or destroyed. The new organ sometimes perfectly resembles the one which was lost; but in many cases it differs from it in the number of its parts, which are mostly fewer than natural, though in some cases they are augmented. Thus Otto says, that when the tail of a lizard is reproduced the new vertebræ are generally destitute of processes; and St. Hilaire has observed a deficiency of the eminences and horny plates with which the tail is furnished in many of these reptiles. In the lobster and other crustacea, on the contrary, when a claw is reproduced, it is frequently furnished with some supernumerary parts. The duplication and even the multiplication of the tail of lizards, and particularly salamanders, may be artificially produced by cutting off this organ, and dividing the stump into two or more strips, each of which, if kept separate during the process of healing, will elongate and form a perfect and distinct tail.

Supernumerary vertebræ have often been observed in the dorsal and lumbar regions; but we are only acquainted with one authentic case in which the number of cervical vertebræ was altered. Leveling once met with eight in an adult man. This constancy in the number of cervical vertebræ is very remarkable, especially when compared with the zoological fact that no mammiferous animal is known to possess more than seven of these bones.

One of the most common anomalies, by increase of parts, is the addition of supernumerary mammæ. The frequency of this malformation may be referred to the circumstance that, in almost all the mammalia, several of these glands exist which are disposed in two parallel series. Man, then, being provided with but two, forms an exception to the general plan; but by the addition of more, the normal arrangement in other mammalia is represented, and the series of these organs established. In anomalous cases, the most frequent number is three, but four and five mammæ have been met with. When four exist, they are generally arranged symmetrically, two on each side of the chest; when three or five are seen, the odd one may be placed laterally, beneath one of the others, or in the median line; in the latter case it is always small and rudimentary, which imperfect development St. Hilaire supposes to arise from the arrangement of the mammary arteries being lateral, so that the gland, when placed in the median line, will receive but a limited supply of nourishment. A very remarkable but rare anomaly is the existence of a mamma in the inguinal region: a case of this kind was observed by Dr. Robert in a woman whose mother was furnished with supernumerary breasts. The organ was here placed on the external part of the left thigh, four inches below the great trochanter. Until the time of pregnancy this mamma was taken for a simple nævus, but at this period it began to develop, along with the natural breasts. It acquired the size of half a citron, and secreted milk; and the author says that the child sometimes sucked the inguinal mamma, and sometimes the thoracic. (*Journal Gén. de Méd.*, t. c. p. 57.)

The second group into which our author has divided the various malformations of the animal body is that of ***heterotaxies***, or general transposition of organs. The peculiar characteristic of this group is that the anomaly affects at the same time a great number of organs, or, in other words, is complex, and yet does not interfere with the accomplishment of any function; the first circumstance distinguishes it from simple anomalies, and the latter from true monsters. In the splanchnic inversion (the only kind that can occur in man and other symmetrical beings), all the viscera, both thoracic and abdominal, double or single, have exactly an opposite arrangement to that which constitutes their natural state. All those commonly situated on the right side, as the liver, cœcum, &c. are found on the left, and *vice versâ*, so that the whole of the contents of the splanchnic cavities present exactly the same appearance as the organs when in their natural situation would do if reflected from a looking-glass. This peculiarity of arrangement is so far from producing any inconvenience to the person affected with it that, in almost all the cases which have been observed, the anomaly has not even been suspected during life; and to prove that it does not tend to shorten existence, we may mention the celebrated case of the invalid soldier, in whom this malformation was discovered after death by Moraud, in 1660, and communicated by Méry to the Academy of Sciences. This man lived to the age of seventy-two, and had pursued a laborious occupation.

The mode of origin of this anomaly is exceedingly obscure; and the causes which give rise to it cannot be understood, until we become acquainted with the laws which govern the normal arrangement of parts. St. Hilaire has adopted a fanciful hypothesis, which was proposed by M. Serres, who says that some one organ regulates by its development the situation of all the others. The organ selected for this purpose is the liver, which certainly seems to perform a more important function in fœtal than adult life. In the early stages of the embryo this organ is large, symmetrical, and placed like the heart in the median line. Subsequently, by the unequal development of the two lobes, it is pretended that the arrangement of all the other viscera, both thoracic and abdominal, is determined. When the left lobe contracts, as in normal cases, the aortic side of the heart, the spleen, large end of the stomach, &c. are drawn to the left side, and the small extremity of the stomach, the pulmonary side of the heart, &c. go to the right; if anything alters the mode of development of the liver, the other organs take a contrary position. We confess that we cannot allow to this hypothesis any other merit besides that of ingenuity.

We have already remarked that in those animals which have unsymmetrical bodies, as some fish and many mollusca, the inversion is general, and extends to all the organs, both external and internal; these cases, however, possess no points of peculiar interest, all the phenomena being of the same character as in the partial instances: we shall, therefore, proceed to the next group, which contains the different forms of hermaphrodism.

"An hermaphrodite," says St. Hilaire, "according to the literal meaning of the word, is a being possessed of both sexes, and either able to fecundate itself or alternately to impregnate others, and to be impregnated; two modes of generation, numerous examples of which may be observed within the limits of the animal king-

4286

dom. The term hermaphrodite was formerly employed in this sense in teratology, when applied to man. The ancients designated by this name certain individuals, to whom they attributed the marvellous faculty of fulfilling by turns the reproductive functions of both sexes, or at least who simultaneously possessed both the male and female organs fully developed." (Tom. ii. p. 30.)

In modern times, though the term hermaphrodism has been retained in compliance with ancient custom, its signification has been much extended, and it is now used to designate not only an individual in whom both sexes are united, but also one who possesses any mixture of the two characters. St. Hilaire divides this group into two classes: one of which is with, and the other without excess in the number of parts. Thus, in other words, this anomaly may result from the union (always more or less incomplete) of the organs of both sexes in the same individual, some of the parts of one being added in excess to the reproductive apparatus of the other; or the hermaphrodism may consist in the possession of only some of the characters of both sexes, the organs remaining essentially single, but having some parts which resemble the male, and others the female sex. These two classes may be again subdivided into several orders. In hermaphrodism without excess, the generative organs may be essentially male or female, a few parts only presenting the opposite sexual conditions; thus arise the two orders of *masculine* and *feminine* hermaphrodism, which were determined by Ambrose Paré. The sexual apparatus, on the contrary, may present such an association of the two genders, and their characters may be so combined, that the determination of the true sex is either difficult or entirely impossible. This may result from two kinds of modification, which have generally been confounded, and of which St. Hilaire constitutes two separate families; in one case the sexes may be so combined that the organs are really neither male nor female, but may be called *neuter;* while in others the characters are so divided that one half of the sexual organs are decidedly female, and the other male, when they are called *mixed.*

Hermaphrodism with excess in the number of parts may also be divided into several orders. St. Hilaire makes three: thus, if a few female parts are added to a perfect male being, *complex masculine* hermaphrodism is constituted; if, on the contrary, a female possesses some supernumerary male organs, we have *complex feminine* hermaphrodism; thirdly, if the organs of both sexes are combined in the same subject, the order of *bisexual* hermaphrodism is formed, which may be compared to the simple mixed order of the former class. If the characters of both sexes were complete in the last order, the formation of a perfect hermaphrodite would result; but though many such cases are recorded by the older writers, they cannot be considered as authentic, and the existence of such a being among the higher classes of animals must be denied.

The mode of production of hermaphrodism without excess has been explained by the relation existing between the male and female organs of generation; which relation or resemblance becomes very close if we compare them together in the young embryo.

"There is one period in the life of the embryo," says M. St. Hilaire, "when all the parts appear female, and another in which they seem to be all male; so that the similitude is thus complete between the sexes. This anatomical analogy between the male and female organs, which was suspected by Aristotle and Galen, and declared by Buffon and many other authors, is now perfectly established, both by the zootomical researches of my father and M. Blainville, and also by the embryological observations of Ferrein, Autenrieth, Home, Ackerman, Meckel, Burdach, Tiedemann, and Serres. It will be found the key to all the anomalous states, which constitute hermaphrodism without excess, and which are inexplicable by any other hypothesis. In fact, if each part of the male apparatus is essentially analogous in its elementary composition to some part of the female, if their apparent difference only results from some variation in the mode or degree of their development, nothing can be more easy to conceive than intermediate states between the two extremes, between those two opposite forms which constitute the normal states of either sex. If, for example, the clitoris be considered as a penis arrested in its development, and, on the contrary, the penis as an hypertrophied clitoris, if in fact the one is the first, and the other the last stage of evolution of organic elements, which are perfectly analogous, we see that all excess of development of the one, all default of the other tends to make them fall into those conditions which are intermediate between the natural states of both. Is it not clear that if this excess or defect of development be carried to a great extent, the clitoris may be changed into a true penis, or the penis be reduced in its composition to the form and volume of a simple clitoris? Thus in the midst of other parts essentially male, a female organ may be found, and *vice versâ*, and that mixture of the sexes may arise which was considered as a prodigy by the ancients, but which is now known to be only the natural result of an excess or arrest of evolution in certain organs."* (Tom. ii. p. 44.)

Another principle by which our author accounts for many cases of hermaphrodism is the division of the sexual organs into six principal segments, which, he says, arise from distinct centres of formation, and which are, to a certain extent, independent of each other; these six segments, three of which belong to each side, are divided into deep, middle, and external.

"The two deep segments are formed by the ovaries or testicles, and their appendages, the middle ones by the uterus or prostate and vesiculæ seminales, &c., and the external by the clitoris and vulva, or penis and scrotum. We cannot fail to observe that these six segments correspond to six different sets of vessels; the deep organs are nourished by the spermatic arteries, the intermediate ones by the hypogastric, and the outer segments are supplied by the external pudic, receiving some branches as well from the hypogastric." (Tom. ii. p. 50.)

This independence of the different portions of the sexual apparatus, may be shown by many examples; thus it is not uncommon to see one segment undergoing modifications in form or structure, and varying even in existence without affecting the other segments. An individual, for instance, may have the deep-seated organs masculine, while the middle ones are feminine; he will then possess testicles and an uterus. The external parts in this case generally partake of the characters of both sexes, but not necessarily.

Our limits will only allow us to describe briefly the different orders of hermaphrodism.

In the variety termed *masculine*, the organs are essentially male, but undergo some modification which produces a greater or less resemblance

* Dr. Robert Knox, of Edinburgh, states that the sexual organs of the embryo, instead of being at one period male, and at another female, or, as Meckel says, always originally female, are in the early stages of formation, neither one nor the other, but intermediate or hermaphrodite; and he explains the origin of monsters belonging to this group by the occurrence of simple arrest of development.—*Med. Gaz., Jan.* 1839.

to the female sex; thus, the penis may be small, the glans imperforate, the urethra changed into a simple furrow, or altogether obliterated, the testicles imperfectly developed, and the scrotum frequently cleft into two portions, which more or less resemble the labia. The urethra commonly opens into this cleft, which, when deep, may be taken for the vagina; the testicles, in some of these cases, remain in the abdomen till a late period, and may even not descend into the scrotum at all, which will sometimes make it very difficult to determine the true sex. Together with these local characters, the general appearance of the body is mostly altered, and has a feminine character; the breasts are developed like those of a young woman, the skin is soft, and the beard thin or wanting.

*Feminine* hermaphrodism is marked by characters the reverse of masculine: the clitoris is mostly very much enlarged, and more or less resembles a penis; it may even be furnished with an imperfect urethra, running along its under surface; the vulva is contracted or imperforate, and in some cases the ovaries descend through the inguinal canal into the labia. Together with these alterations, some of the other characters of the opposite sex are generally present, as a deep voice, masculine form, small breasts, &c.

In these two orders the deep or formative organs, as the testicles, prostate, &c. in the one sex, and the ovaries, uterus, &c. in the other, preserve their natural structure, and thus the being is essentially male or female; yet the external parts and general aspect of the body are often so modified, that it becomes difficult, in some cases, to detect the real gender.

Complete *neuter* hermaphrodism, in which all the organs should offer such an equal degree of development between the two sexes that their real nature cannot be determined, even by an anatomical examination, is very rare; partial cases, however, are often met with, the true sex of which it is often very difficult to determine by an external examination; but if carefully dissected after death, they may mostly be referred to one of the preceding orders.

The best marked examples of neuter hermaphrodism have been observed in animals. Sir Everard Home has described a dog which possessed a well-formed vulva, and a large clitoris or imperforate penis, beneath which was an opening leading to the urethra. These external organs seemed to indicate that the animal belonged to the female sex, and so it was considered until the internal parts were examined, when it proved to be neither male nor female. The reproductive organs consisted of a portion of elongated ligamentous substance, which Home considered as an imperforate vagina, of two small solid cords, intermediate in structure and disposition between vasa deferentia and round ligaments; and lastly, of two ovaries or testicles of a very equivocal nature, to which the cords mentioned were attached. These bodies, which Home considered as testicles, were very small, imperfectly formed, and of a doubtful structure; they occupied the position of ovaries. Home has added to these details that this dog never showed any sexual inclinations or heat, and possessed neither externally nor internally any vestiges of mammæ.

*Mixed* hermaphrodism consists in the union of distinct parts belonging to opposite sexes in the same set of generative organs. This anomaly may be varied in different ways, as either the deep organs may belong to one sex and the superficial to another, or all the segments on one side moy be male, and on the other female. This order must be explained on the principle, which we have already mentioned, of the different parts being separately developed, and independent of each other at the first period of their formation. For the explanation of the latter variety of mixed hermaphrodism, denominated lateral, in which the organs of one side belong to one sex, and those on the other to the opposite, an ingenious hypothesis has been proposed, viz. that it is the product of the intimate union or fusion of two individuals of opposite sexes, one half of each of whose bodies has become atrophied, and in whom the division of the sexes remains as the only vestige of the original duplicity. This idea, though ingenious, is not borne out by any facts.

Hermaphrodism with excess in the number of parts is characterized by the union of two sexes, having separate sets of sexual organs, which may be more or less complete, so that this class presents many degrees; thus to the male apparatus a few only, or nearly the entire set of female parts may be added, and *vice versâ*, giving rise to the two orders of *complex* masculine and feminine hermaphrodism. The third order of *bisexual* hermaphrodism consists in the coexistence of two nearly perfect generative systems of different sexes in the same individual. A most remarkable instance of this anomaly has been recorded by Schrell, a German anatomist. (Vide *Med.-Chir. prakt. Archiv. von Baden*, &c. t. i. 1804.) In this case there existed beneath a true penis, and independently of the testicles and vasa deferentia, which were naturally formed, a small vulva, furnished with labia and nymphæ, and communicating through a true vagina with a rudimentary uterus, provided with round ligaments and imperfectly developed ovaries. Here the two sets of organs were nearly complete, but the male parts were fully developed, while the female remained in a rudimentary state. This case is exceedingly interesting, from being the only authentic instance of the kind that has been met with in man.

Perfect hermaphrodism, where both sets of organs shall be fully developed, is not only unknown among the authentic details of anomalies, but is physically impossible, without great alteration of the natural connexions of the bones and other parts of the pelvis.

We now come to the last and most important group of anomalies, the monsters properly so called, which group includes all the complicated and extreme states of malformation. We shall here find the same facts and same phenomena governed by the same laws as in the more simple anomalies which we have already passed in review.

Zoologists have long since distinguished in the animal series some beings which they call simple, and others which are formed by the aggregation of two or more of the former, which they denominate compound. In the same manner there exist among monsters some which only possess the complete or incomplete elements of a single individual, while others are composed of the parts of two or more beings, united together. Thus St. Hilaire forms the two classes of single and compound monsters. The former class is again subdivided into three orders, as follows:

1. The first comprises those beings which are capable of living, and deriving nourishment by their own proper organs (*autosites*). These can all exist for a longer or shorter time, after leaving the uterus of the mother; and the malformation only affects one part or region of the body, the others preserving nearly their natural state; the circulating system is more or less perfect, and especially the heart; the lungs, most of the digestive organs, and some part of the head are constantly present.

2. The second order (*omphalosites*) includes those monsters which only possess an imperfect and passive kind of life, sustained by means of the communication with the mother, and ceasing when the umbilical cord is divided. These monsters want a great number of organs, and have those which remain in a very imperfect state; all the external regions of the body are deeply malformed, the symmetry of the two halves is imperfect, and often entirely lost.

3. The third and last order of simple monsters is that of parasites, which are the most imperfect of all beings, consisting of irregular shapeless masses, principally formed of bones, teeth, hairs, and fat; they even want the umbilical cord, and are fixed immediately to the generative organs of the mother, at whose expense they lead an obscure and vegetative existence.

Compound monsters are divided into double and triple, each of which subclasses contains two orders, named *autositaires* and *parasites;* the former of these includes those monsters which are composed of two or more individuals, offering the same degree of development, and contributing to the maintenance of the common life. A great many genera are contained in this order, some consisting of monsters completely double; others of those which are only half double, or even single in the greater number of parts.

The second order, that of compound parasitic monsters, contains those beings which are composed of two or more very unequal and dissimilar individuals, one being nearly or quite complete, and the other very small and imperfect, and parasitically attached to the first, of which it forms a mere appendage.

To revert to the subject of *single monsters.*

"The recent progress of embryology," says M. St. Hilaire, "enables us to distinguish three successive stages or phases in the life of the embryo, which are of very unequal duration; the first is very short, in which the embryo, scarcely formed, is fixed directly to the walls of the uterus. In the second, the embryo is distinctly shaped, and is furnished with an umbilical cord. In the third and last stage the embryo or fœtus, as it is now mostly termed, is completely developed, and capable of leading an independent existence by means of its own organs. These three stages evidently have their representatives in the three orders of single monsters, the two most imperfect of which arise from arrest of development taking place at a more or less early period, and to a greater or less extent." (Tom. ii. p. 196.)

Those simple monsters which are capable of supporting an independent existence, may be divided into several tribes and families.

1st. The trunk may preserve its natural form while the limbs are greatly altered; thus they may be entirely suppressed, or two of them may unite into one, as in those monsters termed *symeles* or sirens.

2d. The body may be malformed, and the limbs nearly natural. In this family extensive congenital hernia, or eventration of a great number of viscera, is generally observed. The head both in this, and the former tribe, is slightly, or not at all altered, while in the subsequent divisions it is the organ principally affected.

3d. In the third tribe the face is natural, or only slightly altered, while the cranium and brain are greatly malformed. Thus the brain may exist in a more or less incomplete state, but be situated partly or wholly without the cranial cavity, the walls of which are imperfect. In some cases the brain is entirely deficient, its place being partly occupied by a bright red coloured tumour, which is composed of a number of small vessels. This body lies on the base of the cranium, the upper part or vault of which is nearly all wanting. In other cases the whole brain and cranial arch are undeveloped, and this vascular tumour is not found.

4th. In some monsters the face is more extensively deformed than the cranium. In these cases there is generally atrophy of some of the central facial organs, and approximation or union in the median line of some of the lateral organs, as the eyes and ears.

We shall make but a very few observations on the monsters belonging to this order.

In those named *celosomi*, in which the trunk is malformed, we have remarked that eventration, or displacement of the viscera, constantly occurs; and it has been observed that this malformation realizes, with some modifications, those organic conditions which naturally exist in the early periods of uterine life. In fact, it may represent any of the stages through which the embryo passes, from the primitive state, when all the viscera float loose and are contained in the sheath of the umbilical cord in front of the yet open cavity of the abdomen, to the last period in the formation of the fœtus, when the abdomen is completely formed and closed, except at the point where the umbilical cord is attached. In some of these monsters the funis itself is very short and imperfect, so that the fœtus is fixed close to the placenta; and is, consequently, placed against the walls of the uterus. St. Hilaire is of opinion, that the movements of the limbs may be thus impeded, and deformity of them occasioned, such as is frequently observed in these cases.

If we examine the red tumour which is found occupying the situation of the brain in some cases of malformation of the head, we shall find that it is composed of three parts; first, of vessels which form the constant and principal bulk of the tumour; second, of a collection of serous fluid, which is generally but not always present; third, of vestiges of nervous matter, which are only met with in a few cases. The body thus formed is doubtless the result of atrophy of the brain, and of excessive hypertrophy of the pia mater, and of the intercranial or meningeal vessels, which, instead of being dispersed on the surface, and in the interior of the cerebral organs, become agglomerated into a considerable mass, and form a sort of brain composed wholly of vessels.

Teratology throws considerable light on, and is able in some cases to clear up disputed points in physiology. It has been said that the cerebro-spinal axis is the sole seat of life in the higher animals. It is, therefore, interesting to enquire, whether those monsters which are deprived of the greater part or the whole of the brain and spinal cord, as in the *anencephali*, whose nervous system is reduced to the state met with in insects and worms,* can support an independent existence or life after being separated from the mother.

"All doubts on this subject," says our author "have been long removed by the record of many authentic facts. The first monster of this kind which was met with by M. Vincent Portal, lived a quarter of an hour, and was violently convulsed; it would probably have existed longer had it not been dropped by the nurse. In the case of M. Fauvel, life was prolonged for two hours, with evident signs of sensibility. The monster observed by M. J. J. Sue moved in several directions, and lived for seven hours; that of Malacarne for twelve, and the one of Méry for twenty-one hours, and took some nourishment. Lastly, this is not the longest period to which life may be prolonged in these imperfect beings; for an anencephalous fœtus, born in 1812, at the Hôtel Dieu at Paris, and which was seen by M. Serres, then inspector-general of this hospital, lived three days, and was fed with milk and *eau sucrée*, no nurse being found willing to suckle it." (Tom. ii. p. 371.)

"In reviewing these facts we may observe, that in those monsters which are destitute of brain and spinal cord, as well as in others of the same family in which the brain only is deficient, the spinal marrow existing; during intra-uterine life the malformation does not exert any injurious influence on the development of the embryo; these monsters, up to the time of birth, are strong and healthy; but when they are transported into an external world, which is not adapted to their mode of organization, and are obliged to respire atmospheric air by lungs which are not influenced by the action of nervous centres, they languish and quickly perish. In the same manner as a healthy fish, taken out of the water, dies of asphyxia, in a medium which, though it animates us, is deadly to it: in the same manner also as an embryo dies which is born long before its natural time; so the anencephalous fœtus is necessarily doomed to a more or less speedy death, not because its organization is essentially vicious, but because being only fitted for the conditions of intra-uterine life, it is not capable of supporting a free and independent vitality." (p. 372.)

The cases related by St. Hilaire of anencephalous fœtuses (he includes under this denomination those monsters which are entirely deprived of brain and spinal marrow), which have lived and breathed, even for several days, are exceedingly interesting, if their perfect authenticity may be relied upon; if it was clearly ascertained that no portion of the brain or medulla oblongata existed. For it has always been considered by physiologists that the function of respiration depends on the presence of the medulla oblongata, from which the pneumogastric nerves arise; and it is not easy to conceive how this function can be performed, even for the shortest period, without its influence. It is possible that in these cases some portion of the cerebro-spinal axis was still present, though much displaced and altered.

The second order of simple monsters, which includes those that are altogether incapable of living out of the uterus of the mother, (*omphalosites*,) has been divided into two tribes or families. In the first, the head is nearly or entirely wanting, and the body much malformed and irregular, but yet showing some tendency to preserve a symmetrical form, and inclosing the viscera. The genera *paracephali* and

* The analogy is here not correct: see note, p. 6.

*acephali* belong here, the former of which shows some vestiges of the head, while, in the latter, it is entirely deficient. The other tribe, which contains the *anidiens*, is little known; the body here is still more imperfect than in the former tribe, and no longer includes the viscera, being almost reduced to the state of a cutaneous sac.

The last order of simple or more properly speaking, single monsters, the *parasites*, contain a much greater number of cases, though it is still less understood than the former order. The objects referred to this family are generally called moles, and consist of different organic parts, as teeth and bones, united into an irregular and shapeless mass, which may be found in the uterus, ovaries, or even in other situations. The history of these singular productions is very obscure, and their origin cannot be completely understood; but it seems in some cases, at least, that these parts developed in the uterus or ovaries are the products of conception which have remained exceedingly imperfect, new beings which have commenced under, or at a very early period of their formation have been subjected to, anomalous influences which have drawn them in an unnatural direction. One objection to this mode of explanation is, that tumours containing bones, teeth, hair, &c. have been found in the ovaries of virgins, and of girls before puberty; but these cases, which are very different from the preceding, evidently belong to another class, that of monstrosity by inclusion, and must be placed among the compound monsters which we shall next consider.

We have already stated that double monsters may be divided into *autositaires* and *parasites*. The first of these divisions includes several tribes and families. Thus the two subjects which compose the monster may be both nearly perfect and distinct, only adhering together by one region of the body, and having the component parts of each almost complete even at the point of junction. As an example of this tribe, we may mention the celebrated case of double female, which was born in Hungary in 1701, and which was christened by the two names of Helen and Judith. This monster was shown about for seven years in almost all the countries of Europe, and lived to the age of twenty-two years: the best description of it is given in the Philosophical Transactions, by Torkos. The two individuals were here situated back to back, and united by the buttocks and part of the loins. The external organs of generation offered evident signs of duplicity, but there only existed a single vulva, situated inferiorly and hidden between the four thighs; the vagina was at first single, but soon divided into two distinct canals, and all the other sexual organs were distinctly double. In the same manner there existed two distinct intestinal canals, which terminated by a common extremity. The two vertebral columns were also united by the second piece of the sacrum, and they terminated in a single coccyx. The two aortæ and venæ cavæ united inferiorly by their extremities, and thus established a large and direct communication between the two hearts, which produced a community of life and functions, the source of several very interesting physiological and pathological phenomena.

The two sisters were very different in some points of their characters.

One was much larger and more healthy than the other, as well as more intelligent, though they both spoke several languages; they menstruated at different periods, though there was but a common external genital orifice. The desire of passing the fæces was felt by both of them simultaneously. Their mental and nervous systems seemed to have little communication, for one often slept while the other was awake, and one was sometimes occupied in reading or writing while her sister was in a state of repose. Whenever one was ill, the other soon felt so too, and participated in her sister's disease; it was therefore predicted, and it proved to be too true, that the death of one would necessarily destroy the other. At the age of twenty-two, Judith sunk under disease of the brain and lungs; Helen was seized some time after the commencement of her sister's illness with a slight fever, and suddenly sunk into a state of collapse, preserving, however, her intellectual faculties. After a short struggle, she fell a victim to the death of her sister, and they both expired almost at the same instant.

Another well known instance of double monstrosity, analogous to the last in many respects, but differing from it, however, in some particulars, is the case which was exhibited in London in 1829-30, and denominated the Siamese twins. The junction of the two individuals was here by the ventral aspect of the body, and the monster was furnished with a common umbilicus, whereas, in the former case, there were two distinct cords. For the description of this case we must refer the reader to the periodical medical works of 1830: we have given a brief notice of it in our Second Volume, p. 299.

In the second tribe of double monsters belonging to this class, the component individuals are distinct and separate at their pelvic extremities, but more or less intimately united by their upper halves. In some of the genera of this order the two bodies are completely distinct below the umbilicus, and are surmounted by a compound head; or, in other words, by two heads intimately united together, some of the parts of which are atrophied. St. Hilaire calls these *sycephalous* monsters. In other genera, the two bodies which are separate below are furnished with a single and simple head, in which a very few traces only of duplicity can be discovered. These have been named *monocephalous* double monsters.

Our space will not allow us to enumerate any examples of the malformations belonging to this tribe, which have been principally met with among animals.

The third and last tribe is marked by exactly inverse characters to the preceding; the upper half of the body is double, while the pelvis and inferior extremities are nearly or quite single. Many cases belonging to this family have been met with in the human subject, and we may particularly mention one which excited a great interest in Paris in 1829. This double being, which was a female, and called by the double name of Rita-Cristina, was born in Sardinia, and lived to be nearly nine months old. It was carefully examined after death, and the two vertebral columns were found quite separate in their whole length, and a rudimentary pelvis formed of a single bone separated them inferiorly. Another fully-developed pelvis was situated in its natural position, and supported two well-formed abdominal limbs; the ossa innominata were widely separated posteriorly, so as to include between them the two sacra and the rudimentary pelvis; there existed a single bladder, uterus, and rectum, which were common to the two subjects, but behind these organs were found rudimentary traces of others. There were two distinct hearts, and all the other thoracic and most of the abdominal viscera were double. Many interesting observations were made on this monster during life; the nervous systems seemed to have but little communication, except in those parts which were in the line of union, as the anus and sexual organs; for if the right leg was pinched, Rita only felt it, and if the left, Cristina; thus, of this common pair of limbs, the right seemed to belong to one individual, and the left to the other.* It may be mentioned, that an undeveloped rudiment of a third limb was found in the axis of union of the two innominata, attached to the rudimentary pelvic bone. These two beings experienced the sensation of hunger separately, though they felt the desire to expel the fæces at the same time; this may be explained by the structure of their alimentary canal, which was found to be double as far as the commencement of the ilium, and single in the rest of its course.

The class of double parasites includes those monsters in which one being more or less imperfect is attached as an appendage to the body of another complete individual. In the more highly-developed cases of this anomaly, the accessory being is attached to the external surface of its companion, but in the last tribe the parasite is inclosed and completely hidden within the body of the principal subject.

Monstrosity by inclusion is such a singular anomaly, that it has excited much interest, though at present it is but little understood. The two principal situations in which the inclosed being has been found are in a pouch beneath the skin, or in the abdominal cavity of the perfect individual. In the former of these cases the parasite, though included, is not entirely hidden; for it often forms a considerable projection or tumour in the region where it is placed. The organization of the inclosed fœtus is always very simple and imperfect, yet it may vary very much in this respect; in some cases it may resemble a paracephalous or acephalous single monster, while in others it only consists of a shapeless mass of bones, teeth, and hair, as in the simple parasites named moles. The principal being which contains the parasite is generally well formed in most of its organs, though it is seldom quite perfect in all.

"All the explanations which have been proposed for this anomaly may be referred to five principal heads, viz.: the original inclusion of one ovule within the other, and their simultaneous fecundation; the formation of one ovule from two germs; the inclusion of an ovule or very young embryo in another embryo previously conceived, and already more or less developed; the inclusion of one ovule or embryo in another, which has been conceived at the same time; and, lastly, the production of the inclosed fœtus by the other individual." (Tom. iii. p. 321.)

* Upon dissection, this was found to be the case; the spinal cords were quite separate, and there was no communication between the nerves forming the nervous trunks going to the two abdominal limbs; the only union between the nerves of the two beings, was found in the parts in the line of junction.

Triple monsters are so rare, and so little understood, that there is little to be said about them; their extreme rarity is easily accounted for; the formation of a triple monster requiring two conditions, viz., the simultaneous presence of three embryos in the uterus, and the union of all three together into a compound fœtus. In the human species we know how rare cases of triple birth are, and in those which do occur the children may be disposed in three modes: they may be all separate, as in the normal and common instances; two of them may be united together, the third being free, a disposition which has been observed in a very small number of cases; thirdly, the union of three subjects into a single being constitutes the last of these three modes, and is necessarily the most rare of them, for it results from the coexistence of two monstrous unions, and not of one, as in the former case. Triple monsters have been so seldom seen, that their existence has been doubted by some authors, among whom we may mention Chaussier, Adelon, and Meckel; but St. Hilaire says, that he himself has seen three cases of this anomaly.

The three individuals composing a triple monster are always united in one of the two following ways: they are either all joined together, meeting at the same point, which forms a common centre, or one individual is united to a second, which, placed in the middle, is joined in its turn to a third. These two forms are evidently very distinct, and they have both been realized by authentic observations, which have most of them been met with among animals, in whom this anomaly is not quite so rare as in man.

We must here conclude our notice of M. Is. St. Hilaire's work, which is to be regarded in the light of a compilation, in which the author has most industriously collected a multitude of facts and references, which he has applied to those laws which have been founded or discovered by his father and other naturalists. We earnestly recommend its perusal to all those who take an interest in the higher branches of physiology and philosophical anatomy. The book, however, is not without numerous faults: it has been spun out to an unnecessary length, and is often deficient in clearness of arrangement; the general conclusions, as the laws and causes of anomalies, for instance, though nominally placed together in a distinct section, will be found in many instances scattered through various parts, and can only be collected with difficulty. The pages are often disfigured with those defects which are certainly more common in continental literature than in our own, viz., prosy tediousness of detail and endless repetitions; yet, with all its faults, this work must be looked upon with great satisfaction, as filling up a gap in natural and medical science.

